# Accuracy and spread of nest search behaviour in the Saharan silver ant, *Cataglyphis bombycina*, and in the salt pan species, *Cataglyphis fortis*

**DOI:** 10.1007/s10071-020-01371-6

**Published:** 2020-03-27

**Authors:** Sarah Pfeffer, Verena Wahl, Harald Wolf

**Affiliations:** Institute of Neurobiology, Ulm University, Albert-Einstein-Allee 11, 89081 Ulm, Germany

**Keywords:** Saharan silver ant *Cataglyphis bombycina*, *Cataglyphis fortis*, Nest search behaviour, Odometer accuracy, Precision, Navigation certainty

## Abstract

Desert ants of the genus *Cataglyphis* are renowned for their navigation abilities, especially for their beeline homing after meandering foraging excursions reaching several hundreds of meters in length. A spiralling nest search is performed when an ant misses the nest entrance upon completing its homebound travel. We examined the nest search behaviours of two desert ant species dwelling in different habitats—*Cataglyphis bombycina* living in the dunes of the Sahara and *Cataglyphis fortis* found in the salt pans of North Africa. The two species show distinct differences in walking behaviour. *C. bombycina* performs a strict tripod gait with pronounced aerial phases, high stride frequencies, and extremely brief ground contact times. In view of these peculiarities and the yielding sand dune substrate, we hypothesised that homing accuracy, and namely distance measurement by stride integration, should be lower in *C. bombycina*, compared to the well-studied *C. fortis* with less specialised walking behaviour. We tested this hypothesis in ants’ homebound runs from a feeding site in a linear channel setup. Surprisingly, the accuracies of nest searches were similar in the two ant species, and search accuracy was also independent of the walking substrate, soft dune sand or a hard floor. The spread of the nest search, by contrast, differed significantly between the two species, *C. bombycina* exhibiting a larger search spread. This may be interpreted as an increased path integration uncertainty due to the above locomotor specialisations, or as a compensation strategy accounting for the silver ants’ particular environmental and behavioural situation.

## Introduction

**Fig. 1:**
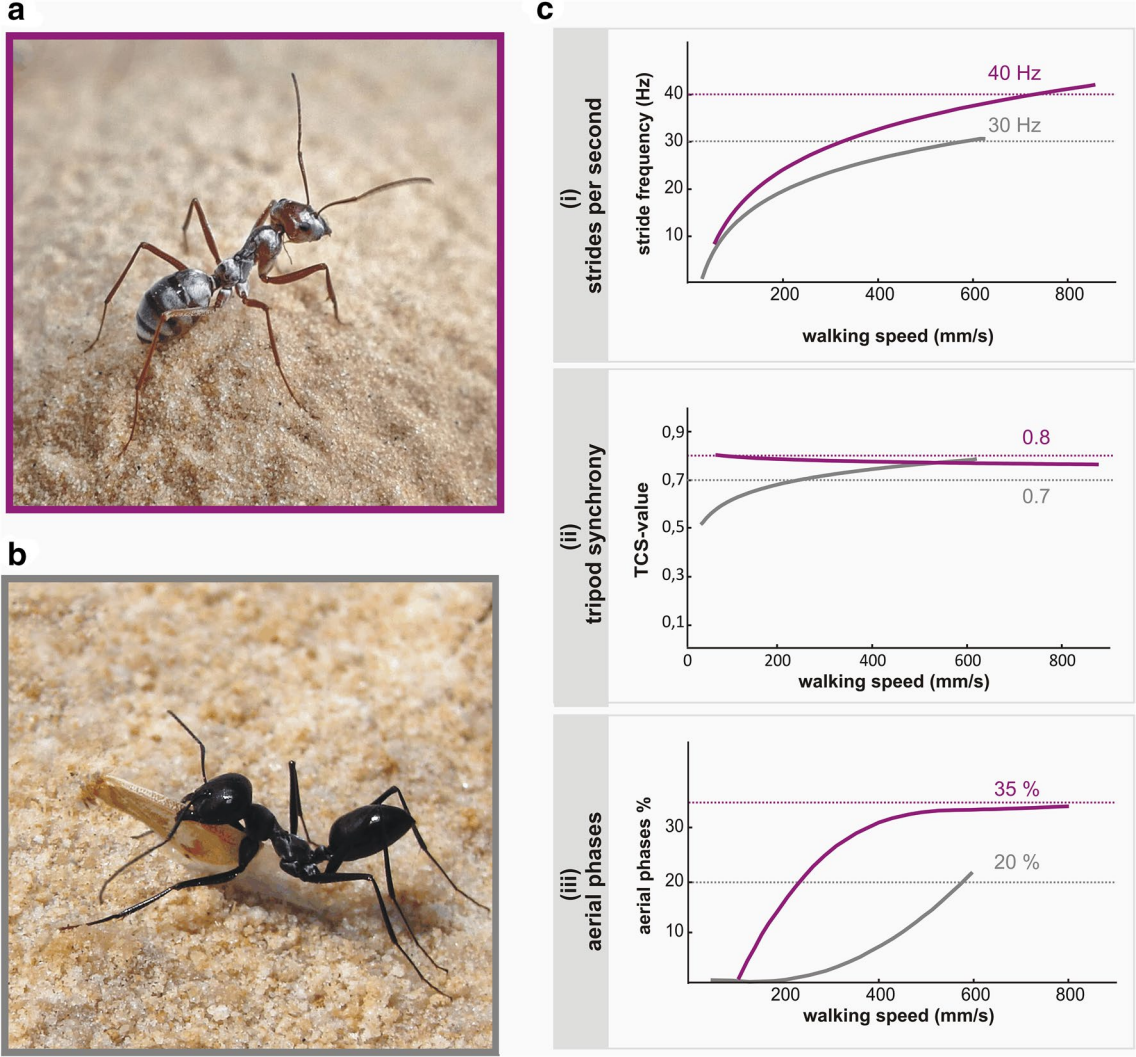
Saharan silver ant (*Cataglyphis bombycina*) and salt pan species, (*Cataglyphis fortis*). **a** The Saharan silver ant, *C. bombycina*, dwells in Sahara sand dune environments. **b** *C. fortis* lives in North African salt pan habitats. Note that image frame colours correspond to colour coding of data for the two ant species presented in this and the following figures. **c** Walking parameters in species-specific comparison for *C. bombycina* and *C. fortis*. The three parameters stride frequency (i, top), tripod coordination strength (ii, middle) (TCS, values of ‘1’ indicating absolute synchrony, the lower the value the less synchronous leg movements in one tripod group (Wosnitza et al. 2013)), and fraction of aerial phases (all six legs off the ground) in % of the step cycle duration (iii, bottom) are plotted as averaged values against walking speed on the abscissa. Dotted reference lines are meant to facilitate comparison of the two species. Data adapted from (Pfeffer et al. 2019) (colour figure online)

The Saharan silver ant, *Cataglyphis bombycina* (Fig. 1a), is known as the fastest of the North African *Cataglyphis* desert ants (Pfeffer et al. 2019; Wehner 1983) and it is probably the fastest runner among all ant species examined, reaching absolute walking speeds of 855 mm s^−1^, which is roughly 106 body lengths per second. The running performance of the silver ant was recently analysed in detail and compared to that of *Cataglyphis fortis* (Fig. 1b) as reference (Pfeffer et al. 2019). Regarding navigation behaviour, *C. fortis* is arguably the best studied desert ant to date (Wehner 2019), with the Australian *Melophorus* species right after (e.g. Cheng 2012). *C. bombycina* inhabits the sand dunes of the Sahara, while *C. fortis* dwells in the North African salt pans. The two species are closely related but differ in habitat and walking behaviour, with these two aspects perhaps related (Pfeffer et al. 2019).

*Cataglyphis bombycina* ants reach remarkable running speeds despite their relatively short legs, at least by the distinctly long-legged *Cataglyphis* standards, *C. fortis* being a suitable example here (Sommer and Wehner 2012). A detailed analysis of walking behaviour (Pfeffer et al. 2019) revealed several differences between the two species. The three most significant differences are the following.

First, silver ants achieve extremely high stride frequencies during fast runs, with an average of 40 Hz, while maximum values were recorded at 47 Hz (Fig. 1c, (i) top). The short legs, compared to *C. fortis*, may be an advantage here due to the resulting low inertial momentum, allowing rapid alternating leg movements. Ground contact during the stance phase is typically brief and may be as short as 7 ms. The salt pan species *C. fortis*, by comparison (Fig. 1c, grey graphs), has some 22% longer legs, and it reaches lower maximum stride frequencies of an average 30 Hz, with a recorded maximum of 36 Hz.

Second, *Cataglyphis* ants (like many other insects) use tripod coordination during walking, where three legs are more or less coupled in their movements. The front and hind legs on one side of the body move together with the middle leg of the other side, and this tripod alternates with the respective contralateral legs. A characteristic in silver ants’ walking is the high synchrony of the three legs already at the lowest walking speeds. That is, the three legs in a tripod touch down and lift off at almost the same time. Tripod coordination strength (TCS) (Wosnitza et al. 2013) ranges around 0.8 throughout the speed range of 60–855 mm s^−1^ (Fig. 1c, (ii) middle). The strict tripod coordination together with the brief ground contact times were hypothesised to reduce sinking of the tarsi into the soft dune sand (Pfeffer et al. 2019). *C. fortis* ants exhibit less synchronous swing movements of their legs in a tripod (TCS below 0.7) up to intermediate walking speeds, and only at the highest speeds they show TCS values reaching 0.8.

Third, *C. bombycina* extend their stride length by inserting aerial phases between lift off in one leg tripod and touch down in the complementary tripod (Fig. 1c, (iii) top), a locomotor mode related to galloping in quadruped animals. Aerial phases are used by *C. bombycina* already at the lowest walking speeds, while *C. fortis* starting ‘galloping’ only at intermediate speeds. There is a gradual transition towards the highest running speeds where aerial phases occupy about 30% of a step cycle in *C. bombycina* and about 20% in *C. fortis*.

Several aspects of the walking behaviour described above for the silver ant appear to suggest that navigation errors are more pronounced in *C. bombycina* than in *C. fortis*. This concerns namely odometry by stride integration that is used for path integration in these capable navigators (Wittlinger et al. 2006, 2007). The yielding sand dune substrate would be expected to increase slip and provide unreliable leg support, making measurement of stride length undependable. The same would appear to be true for the aerial phases used to extend stride length. Extremely high stride frequencies and short ground contact times would not appear to facilitate stride integration either, although potential problems are less clear than for sand substrate and aerial phases. We would thus expect the odometer to have higher accuracy on the hard floor, and perhaps either species performing better on its typical walking substrate. That is, if odometer performance is adapted to the ant habitat, *C. bombycina* might exhibit best odometer accuracy on dune sand, while *C. fortis* should err more on the dune sand substrate.

To address these assumptions, we compared the nest search performances of the silver ant *C. bombycina* to that of *C. fortis*. Nest search behaviour has been studied in *C. fortis* routinely for many years (Schultheiss et al. 2015; Wehner and Srinivasan’s 1981; Wehner 2019) and is thus an ideal reference for comparison. We focussed on distance measurement, or odometry, since the skylight compass as the 2^nd^ component in path integration is not expected to differ between the two species (e.g. Wehner 2003).

## Materials and methods

### Animals and experimental sites

Saharan silver ants (*Cataglyphis bombycina*, ROGER 1859) and salt pan desert ants (*Cataglyphis fortis* (FOREL 1902)) were observed in their respective habitats. During the summer months of 2016, experiments with *C. bombycina* (Fig. 1a) were conducted near Douz, Tunisia, (33.44°N, 9.04°E), while the experiments carried out with *C. fortis* (Fig. 1b) were performed near Maharès, Tunisia, (33.32°N, 10.33°E). The research project was conducted in compliance with current laws, regulations and ethical guidelines of the University of Ulm and of the countries of Germany and Tunisia. The experiments were covered by a research permission of the national authorities (Ministère des eaux et forêts) and kindly approved by the owner of the land.

### Experimental setup

**Fig. 2:**
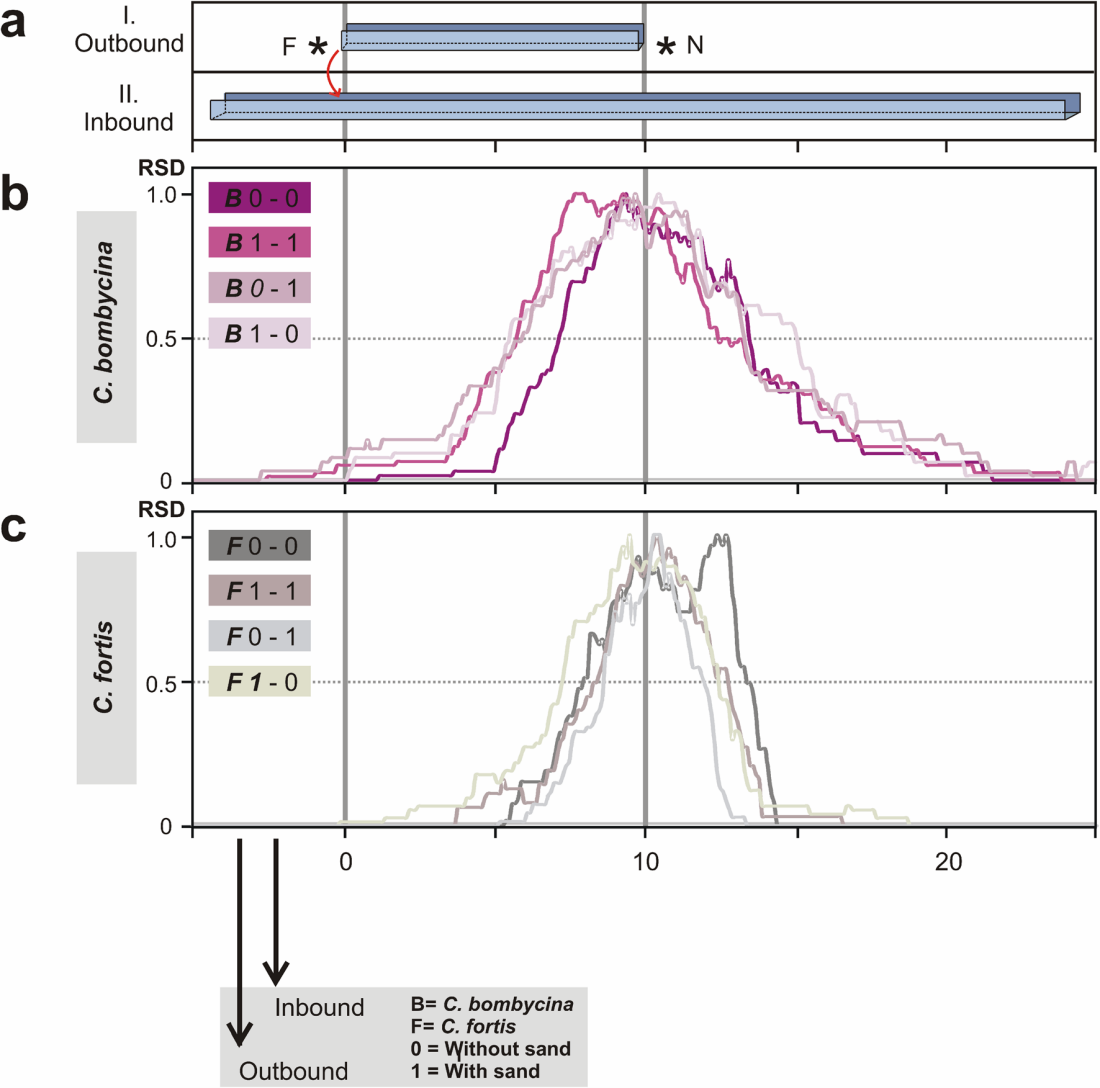
Relative search densities (RSD) in *C. bombycina* (purples) and *C. fortis* (greys) **a** Set-up of outbound training (I.) and inbound test (II.) channels. After an outbound journey of 10 m in the training channel from nest (N) to feeder (F), the ants were transferred (red arrow) to the parallel test channel, where their homing behaviour towards the (fictive) nest was recorded. **b** Relative search densities (RSD) of *C. bombycina* in the different experimental conditions (abbreviation below, and Table 1). RSD value ‘1.0’ denotes the most frequently visited segment of the test channel, RSD value of ‘0’ marks parts of the channel not visited at all. **c** Relative search densities (RSD) of *C. fortis* in the different experimental conditions. Dotted line marks 0.5 value of search density distribution. The distance between two points on one graph that crosses the 0.5-value-line is the full half width of respective search distribution. Note that *C. bombycina* shows here larger full half widths than *C. fortis*. In the labels, “***B***” signifies *C. bombycina*, “***F***” *C. fortis*; “0” means that the respective channel was not filled with dune sand but rather had a hard, sand-coated floor, while “1” signifies dune sand substrate; the first number denotes the training channel, the second number the test channel (colour figure online)

Experiments were performed according to established procedures (Steck et al. 2009; Wittlinger et al. 2007). A nest site was connected by a U-shaped aluminium channel (7 cm wide, walls 7 cm high) with a feeder station at 10 m distance. The feeder was equipped with cookie crumbs cut and sieved to about 1.5 mm diameter. A parallel test channel with a length of 30 m was installed to record the ants’ homing behaviour during inbound journeys (Fig. 2a). These channels make quantitative recording of walking behaviour and odometry data much easier compared to the open desert terrain (e.g. Wolf et al. 2012), and the channel floor can be filled with appropriate walking substrates. In the present experiments, the channel was either filled with a layer of desert sand (2–3 cm thick and collected in the neighbourhood of Douz, Tunisia), or the floor consisted of the channel aluminium laminated with sand for traction and reliable tarsal grip. The sand was glued to the aluminium surface in this case, forming a sand paper-like walking substrate.

### Training and test procedures

For individual recognition, ants were marked with car paint applied to alitrunk or gaster. Only individuals that had visited the feeder at least five times previously were tested, thus ensuring that tested ants were familiar with the channel setup. Each ant was used only once for testing. To test the ants’ homing behaviour, they were carefully captured at the feeding station after they had covered their 10 m outbound walk from the nest. The captured animals were released into the test channel as soon as they had grabbed an offered food morsel. Only ants that held on their food item throughout the entire test procedure (proving their motivation to return to the nest) were considered for further analysis. The ants’ search behaviour was recorded by noting their *U* turns in the test channel, that is, an inversion of walking direction maintained for at least 20 cm. While the first turn after straight homing terminates the ants’ determined homebound travel and marks the beginning of nest search (e.g. Cheng and Wehner 2002), six consecutive turning points were recorded to determine the ants’ search distribution and calculate the corresponding medians and variances, the latter serving as proxies for spreads of nest search behaviour. Release point and turning points were determined to the nearest 10 cm with a tape measure strung along the test channel. These 10 cm bins were used in all further evaluations (see below).

Training and test channels were equipped either with sand or with a hard floor in all possible combinations and for the two ant species examined, resulting in altogether eight experimental groups (see Table 1). These eight groups are labelled in text and figure legends below as follows. “***B***” signifies *C. bombycina*, “***F***” *C. fortis*; “1” means that the respective channel was filled with dune sand, while “0” signifies that the channel had a hard floor; the 1^st^ number denotes the training channel, the second number the test channel. ***B ***1–0 thus labels the experiment where *C. bombycina* had reached the feeder via a training channel filled with dune sand and was tested in a channel with a hard floor.

**Table 1:** Test groups and experimental paradigms

Group	Species	# ants	Floor outbound	Floor inbound
***B*** 0–0	*C. bombycina*	*n* = 21	Hard floor	Hard floor
***B*** 1–1	*C. bombycina*	*n* = 22	Dune sand	Dune sand
***B*** 0–1	*C. bombycina*	*n* = 21	Hard floor	Dune sand
***B*** 1–0	*C. bombycina*	*n* = 20	Dune sand	Hard floor
***F*** 0–0	*C. fortis*	*n* = 22	Hard floor	Hard floor
***F*** 1–1	*C. fortis*	*n* = 20	Dune sand	Dune sand
***F*** 0–1	*C. fortis*	*n* = 20	Hard floor	Dune sand
***F*** 1–0	*C. fortis*	*n* = 20	Dune sand	Hard floor

Experimental test groups are labelled either with ***“B”*** referring to *C. bombycina*, or ***“F”*** referring to *C. fortis;* the following two numbers give information about respective floor condition; “1” means the respective channel was filled with dune sand, while “0” signifies the channel had a hard floor; the 1st number denotes the training channel, the second number the test channel

### Data recording and statistics

To evaluate the ant’s search behaviour, we calculated search density distributions from the recorded turning point data. The 10 cm bins of the test channel covered by an ant during its nest search were cumulated, that is, the more often a bin was visited during this search behaviour, the higher was its value in the density distribution. For better comparison, we normalized each search density distribution to its peak value (relative search density (RSD), values between 0 (not visited) and 1 (most visited position), Fig. 2b, c).

**Fig. 3:**
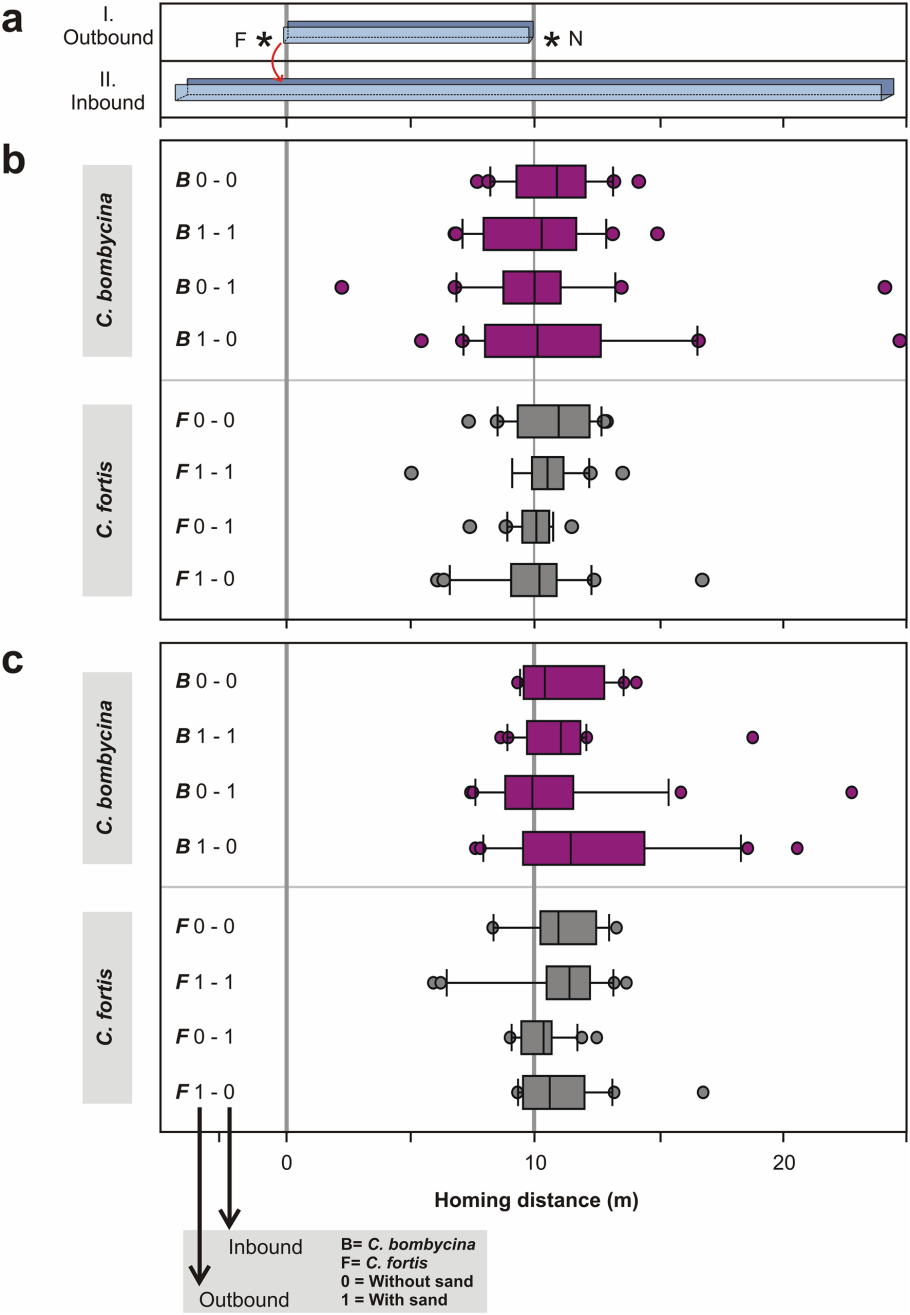
Search accuracy—comparison of nest searches in *C. bombycina* (purple) and *C. fortis* (grey). **a** Set-up of outbound training (I.) and inbound test (II.) channels (compare Fig. 2a). **b** Medians of nest searches calculated from the initial six turning points. There is no statistically significant difference between any of the test groups (ANOVA on ranks (*p* = 0.613)). **c** First turning points of nest searches. There is no statistically significant difference between any of the test groups (ANOVA on ranks (*p* = 0.182)). Neither is there any difference between species, when pooling the data from all experimental situations in each species (comparison of pooled medians: *U* test; *p* = 0.897, comparison of pooled 1st TPs: *U* test; *p* = 0.897). Same data set as in Fig. 2, labelling details as in Fig. 2 (colour figure online)

**Fig. 4:**
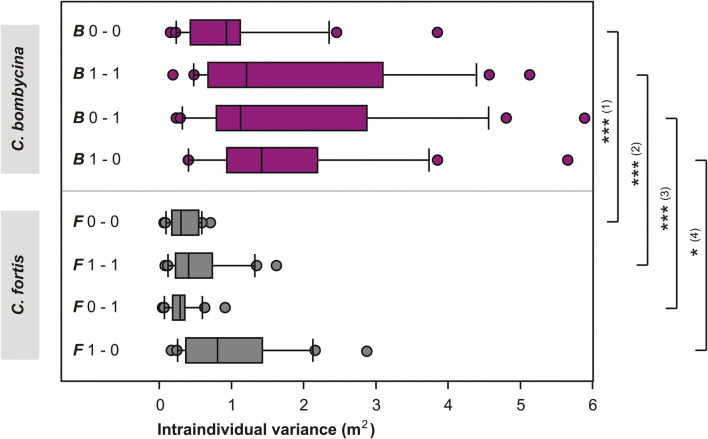
Search precision—comparison of intraindividual search variances in *C. bombycina* (purple) and *C. fortis* (grey). Intraindividual variation (variance of the initial six turning points for each animal) is plotted on the abscissa. Note that there is a statistically significant difference between the different groups (*U* test): (1) ***F*** 0–0 versus ***B*** 0–0, *p* ≤ 0.001; (2) ***F*** 1–1 versus ***B*** 1–1, *p* ≤ 0.001; (3) ***F*** 0–1 versus ***B*** 0–1, *p* ≤ 0.001; (4) ***F*** 1–0 versus ***B*** 1–0, *p* ≤ 0.019; The species-specific difference is demonstrated even more clearly when pooling the data from all experimental groups in a species, *p* ≤ 0.001. Same data set as in Fig. 2, labelling details as in Fig. 2 (colour figure online)

To examine the *accuracy* of an ant’s search, we calculated the search median (Fig. 3b) of the initial six turning points for each homebound run. We further plotted the first turning point (Fig. 3c) for each tested ant as another parameter characterising nest search accuracy. The *spread* of the nest search represents the *precision* (or certainty) of the respective ant about its nest location. To analyse the spread of nest search, we calculated the variance of the initial six turning points (Fig. 4) for each ant (intraindividual variance). We further compared the variances of the first turning points and search medians of each experimental group (interindividual variance). See also subsection below on terminology.

The values of the individuals in an experimental group were used to construct box-and-whisker plots showing grand medians, 25th and 75th percentiles as box margins, and 10th and 90th percentiles as whiskers. Outliers are shown as data points. We determined medians instead of means, because not all data were normally distributed. All experimental groups consisted of different sets of animals and thus were independent. The Mann–Whitney rank sum test (denoted as *U* test below) was used for pairwise comparisons; the ANOVA on ranks (Kruskal–Wallis test) was used for multiple comparisons of not normally distributed data. We tested for a significance level of 0.05, using SigmaPlot 11.0 (Systat Software, San Jose, CA, USA). Levene’s test was used to compare (interindividual) variances (Table 2), the calculation being performed in Excel (Microsoft Corporation, Remond, WA, USA).

**Table 2:** Nest search precision—comparison of (interindividual) variances of (i) 1st turning points and (ii) nest search medians

	(i) Comparisons of variances in 1st turning points			(ii) Comparisons of variances in nest search medians		
	Variances First TPs (m^2^)	Levene’s test	Variances of 1. TPs different	Variances search medians (m^2^)	Levene’s test	Variances of medians different
***B*** 0–0	3.00	*F* value (1.24) < Critical value (2.09)	No	3.2	*F* value (1.26) < Critical value (2.09)	No
***F*** 0–0	2.41			2.58		
***B*** 1–1	4.14	*F* value (1.07) < Critical value (2.08)	No	4.91	*F* value (1.83) < Critical value (2.08)	No
***F*** 1–1	4.37			2.68		
***B ***0–1	11.86	*F* value (3.57) > Critical value (2.16)	Yes	15.41	*F* value (21.05) > Critical value (2.16)	Yes
***F*** 0–1	3.32			0.73		
***B*** 1–0	11.88	*F* value (13.77) > Critical value (2.11)	Yes	18.58	*F* value (3.88) > Critical value (2.11)	Yes
***F ***1–0	0.86			4.79		
***B*** all	7.59	*F* value (2.77) > Critical value (1.43)	Yes	10.15	*F* value (3.79) > Critical value (1.44)	Yes
***F*** all	2.74			2.68		

Note that there are no statistically significant differences between the variances of the first turning points, nor between the nest search medians (medians of initial six turning points) for ***F*** 0–0 versus ***B*** 0–0 and ***F*** 1–1 versus ***B*** 1–1. However, variances in first turns and medians differ significantly for ***F*** 1–0 versus ***B*** 1–0, ***F*** 0–1 versus ***B*** 0–1, and when data from all experiments in a species were pooled (***F*** all vs. ***B*** all)

### Terminology

The terms *accuracy* and *precision* are used here with the following meanings.

*Accuracy* of a homing ant is reflected by the distance between the (fictive) nest position in the test channel and the start of the nest search, or by the median of the ensuing nest search. The start of the nest search is measured as the *1st turning point* that terminates the homebound run. The search median is a more reliable measure since it is calculated from six turning point measurements and thus less influenced by experimental errors such as misreading of distance values along the channel or disturbances of the animal. The term accuracy is also used for the mean distance measured in an experimental group of ants, although mean accuracy would be more appropriate here. Please note that this use of the term accuracy is different from its meaning in some other fields of research.

Search *precision* (or certainty) is reflected by an ant’s spread of nest search. The more focused nest search behaviour, the more precise it is (or the more certain the ant appears to be regarding nest position). In the present study, we analysed *intraindividual variance* of an ant’s initial six turning points to assess odometer precision. Another way to measure precision in odometry is *interindividual variance* of the first turning points of the individuals within one experimental group. This concept can be expanded to the interindividual variance of search medians. Please note that this use of the term spread is thus related to use of the terms precision or certainty in the literature (e.g. Pfuhl et al. 2011; Wehner 2008).

## Results

We recorded homing performances in the silver ant *Cataglyphis bombycina*, and in the salt pan species *Cataglyphis fortis* for comparison, on two relevant walking substrates. The substrates were either dune sand from *C. bombycina* ‘s habitat, or a hard aluminium floor coated with quartz sand for traction of the animals’ tarsi. The hard floor served as a proxy for the hard and level sun-baked clay in the salt pans inhabited by *C. fortis*. Both ant species were examined on dune sand as well as on hard channel floor (Table 1). We would have expected odometer performance to be higher on the hard substrate, and perhaps either species to perform better on its typical walking substrate, with *C. bombycina* misgauging homing distances more on a hard floor, and *C. fortis* erring more on soft dune sand.

Hence, we performed experiments where outbound and inbound travel were on the same substrate, either sand or hard floor. Furthermore, we determined the animals’ homing performances in test channels that had a floor cover different from that in the training channel. That is, ants that had experienced dune sand substrate during the outbound journey encountered hard sand-coated floor during inbound travel, and vice versa.

### Accuracy of search (median of search behaviour)

The ants did not meet the above expectations in any of these experimental conditions. Regarding search density distributions (Fig. 2b, c), it appears that all experimental groups searched for the nest close to the true nest-feeder distance of 10 m. This is illustrated by positions and peaks of the search density distributions; for statistical analysis see Fig. 3. Search medians ranged from 8.90 to 10.97 m, with an average search distance across all experimental groups in the two species of 10.25 m (Fig. 3b). There were no significant differences between any of the homing distances observed in the different experimental conditions (*p* = 0.613, ANOVA on ranks). These values are in perfect agreement with the homing distances observed in the control experiments of several previous studies (e.g. Steck et al. 2009; Wittlinger et al. 2006, 2007; Wittlinger and Wolf 2013). In other words, independent of the walking substrate, and of potentially different substrates during out- and inbound travel, both ant species judged homing distance quite accurately. This result is of particular significance for *C. bombycina* in view of the hypothesis stated in the Introduction. It does not appear that homing distances in *C. bombycina* are less accurate than in *C. fortis*, on principle, as a consequence of *C. bombycina*’s sand dune habitat. Neither was homing compromised for *C. fortis* when walking on dune sand, as an unusual substrate for this species that might be assumed to handicap stride integration for distance measurement.

Evaluating the initial turning points (1st TPs), that is, the start of search behaviour (Cheng and Wehner 2002), instead of the median of the search distribution, corroborated these results (Fig. 3c). Medians of 1st TPs ranged from 9.90 to 11.45 m, with an average across all experimental groups of 10.70 m. There were no significant differences between any of the 1st TP medians (*p* = 0.182, ANOVA on ranks).

### Precision of search (spread of search behaviour)

There was, however, one significant difference between the homing performances of the two ant species. The (*intraindividual*) spreads of the nest searches (see Materials and Methods for use of the term spread) were consistently larger in *C. bombycina* than in *C. fortis* for all experimental conditions. This is again illustrated when comparing the search density distributions in Fig. 2b and c. Statistical analyses are provided in Fig. 4. Differences between the two species with regard to intraindividual search variances were significant for all experimental groups, independent of walking substrate (Fig. 4). Indeed, the differences were highly significant (*p* ≤ 0.001) for all experimental paradigms except 1–0 (outbound journey on sand, inbound journey on hard floor, *p* ≤ 0.019).That is, *C. bombycina* exhibited a broader search than *C. fortis*, on average by a factor of about 2.8, ranging from 1.76 in the 1–0 condition to 3.84 in the 0–1 condition. This was true even where out- and inbound travel were on the hard substrate for both species, a situation where odometry would be expected to be most reliable.

We further analysed precision of odometry *interindividually*, as reflected by the variance of the 1st turning points and the variance of search medians within one experimental group (see Table 2). In this comparison, different substrates during out- and homebound travel (0–1 and 1–0) produced statistically significant differences between *C. fortis* and *C. bombycina*, the latter species exhibiting broader interindividual variances. In contrast, there was no statistically significant difference between interindividual variances regarding the conditions where out- and inbound had the same substrate (0–0 and 1–1). By the same token, in most analysed conditions (see Fig. 4; Table 2), *C. fortis* showed more focussed searches than *C. bombycina*, demonstrating seemingly more precise search behaviour.

## Discussion

According to our present experiments, the accuracies of nest searches were similar in the two *Cataglyphis* species, and search accuracy was also independent of the walking substrate, be it soft dune sand or a hard floor. The latter held even for different substrates during out- and inbound journeys. This indicates that in our straight and level experimental setup with evenly distributed sand layers, where present, odometer accuracy in *C. bombycina* is similar to that in *C. fortis* and remains unaffected by the walking substrate.

The spreads of the nest searches, by contrast, differed significantly between the two ant species. *C. bombycina* showed broader nest searches than *C. fortis* in almost all comparisons, which may be interpreted in two (not mutually exclusive) ways. First, the characteristics of *C. bombycina*’s walking behaviour noted in the Introduction potentially increases navigation uncertainty and thus spreads of nest searches. Second, the broader search behaviour may be interpreted as an adjustment compensating navigation uncertainty in the sense of a goal expansion strategy (see below). These species-specific differences in search behaviour would appear to be genetically determined rather than acquired from experience.

### Nest search accuracy

The same accuracies of both ant species in navigating back to their nests are surprising when considering that the main orientation mechanism is vector navigation in the desert environment (e.g. Wehner 1987), and certainly in our channel experiments (e.g. Wittlinger et al. 2006, 2007), primarily relying on stride integration for odometry (Pfeffer and Wittlinger 2016; Ronacher and Wehner 1995; Wehner 2009; Wolf et al. 2018). It has to be considered, however, that our experimental setup provided almost ideal conditions for reliable odometry. The experimental channels were straight, level and equipped with a homogeneous walking substrate, either sand or hard floor with good grip. This may explain the accurate odometry performance in both species and its independence of the walking substrate, with accuracies well within the error margins reported in the literature (e.g. Müller and Wehner 1994; Wehner 1987; Wittlinger et al. 2006, 2007). For *C. fortis*, correct homing under a number of experimental conditions, including unusual walking substrates such as corrugated surfaces with different corrugation wavelengths and after removal of legs just before homebound travel has been reported (Steck et al. 2009; Wittlinger and Wolf 2013). This resilience of odometry in *C. fortis* makes the present observation much less surprising. Nonetheless, the underlying mechanisms remain as yet unclear.

### Nest search precision

The different spreads of the search behaviours in the two ant species are indeed notable. Intraindividual search variance in *C. bombycina* is on average about 2.8 times larger than the spread observed in *C. fortis*, averaged over all experimental conditions (range 1.76–3.84 times larger). A consistently broader search behaviour may be considered as a consequence of reduced navigation certainty in the habitat. Observation of homing performance in the dune habitat may clarify these assumptions. After all, in their natural environment, the silver ants encounter steep dune slopes, dune surfaces corrugated by the constant desert wind, and sand with vastly different grain sizes and compositions, from near dust to small pebbles. This environment should affect locomotion odometry at least to some extent by causing tarsal slip or irregular walking conditions which may compromise stride integration, the main source of odometry in desert ants (e.g. Wehner 2009; Wittlinger et al. 2006, 2007). Further uncertainties in distance measurement may be caused by aerial phases typically used by *C. bombycina* more regularly than by *C. fortis* (Pfeffer et al. 2019).

In this context, the sometimes larger *interindividual* variances of searches in *C. bombycina* are notable in experiments where out- and inbound travel were across different substrates (0–1 and 1–0 experiments, see Table 2). There were no differences, however, in experiments where ants walked on the same floor during the entire training and test procedure (0–0 and 1–1 experiments). The reason here might be that with similar walking substrates during outbound and inbound journeys, similar odometer errors should accumulate. Along the line of that argument, travel across different substrates (0–1 and 0–1- experiments) may lead to correspondingly different odometer errors that do not cancel out on the round trip. This imprecision is expected to be more significant in *C. bombycina* due to its more error-prone type of locomotion (see above and Introduction).

A particular behavioural property of *C. bombycina* may belong in the context of compensating for reduced navigation certainty in the dune habitat. This is the so-called goal expansion strategy (Wolf and Wehner 2005) apparently used by *C. bombycina* (Wehner 2009). The presence of the nearby goal, the nest entrance, is signalled here by several stationary nest mates contacting returnees with their antennae. These stationary ants are typically of the soldier sub-caste and distributed over an area of a few square meters around the nest entrance. A broader search behaviour of incoming foragers would appear to be favourable in this case since it increases the chance of running into a soldier, if not the nest entrance itself, and thus ascertaining the nearby presence of the nest entrance. In this sense, stationary soldier ants in the nest surrounds serve as additional orientation cues, complementing the few or absent landmarks in sparse desert environment. This behaviour of *C. bombycina* ants could thus compensate navigation uncertainty. Defence against ants from other nests or predators is another, and perhaps the main, function of this behaviour.

More generally, the spread, rather than the accuracy in particular experimental situations, may be adjusted to navigational task and environmental conditions. There are two conditions that have been reported to influence search spread of desert ants [disregarding landmarks and panorama that provide additional navigation cues and thus affect search behaviour (Bühlmann et al. 2011; Cheng et al. 2012; Schultheiss et al. 2013)]. First, search spread varies with the return distance of the foraging ant, larger distances resulting in appropriately broader search distributions (Merkle et al. 2006; Merkle and Wehner 2010), or in larger safety margins in steering towards a goal to avoid lengthy searches (Wolf and Wehner 2005). Second, searches for familiar feeding sites are much more focussed than nest searches (Pfeffer et al. 2015). This implies that search behaviour is determined not just by uncertainty about goal location (Merkle et al. 2006; Merkle and Wehner 2010) but on what a useful time investment may be for finding the goal, and how essential that goal is for survival. The nest as the most important location in an ant’s life would thus appear to justify broad and lengthy search strategies. In this view, the larger spread of the nest searches of *C. bombycina* may indeed be viewed as an adaptation to larger navigation uncertainty in sand dunes, compared to the salt pan habitat. This interpretation would refine the hypothesis put forward in the Introduction, with the initial idea of a generally reduced navigation accuracy in *C. bombycina* walking on dune sand to be rejected.

Species-specific differences in search behaviour have been addressed very rarely so far. Cheng et al. (Bühlmann et al. 2011; Cheng et al. 2006; Narendra et al. 2007; also reviewed in (Cheng et al. 2014)) have compared nest searches and odometry (first turning points) in the Australian desert ant, *Melophorus bagoti*, and the North African desert ant, *C. fortis*. They observed more precise searches in *C. fortis*, which agrees well with *C. fortis* having to rely on path integration more in its barren salt pan habitat than *M. bagoti* in the more cluttered and often steppe-like habitat. Search spread might be a species-specific property, when considering that search distributions vary as a function of home vector length and not outward path length (which they would if they depended on individual uncertainty). Hence, each species seems to have a fixed relationship between home vector length and search spread which can be interpreted as innate species-specific function. This might suggest that search distributions have been tuned by evolutionary processes—rather than by a Bayesian process at the individual level on each foraging run.

## References

[CR1] Bühlmann C, Cheng K, Wehner R (2011). Vector-based and landmark-guided navigation in desert ants inhabiting landmark-free and landmark-rich environments. J Exp Biol.

[CR2] Cheng K (2012). How to navigate without maps: the power of taxon-like navigation in ants. Comp Cognit Behav Rev.

[CR3] Cheng K, Wehner R (2002). Navigating desert ants (*Cataglyphis fortis*) learn to alter their search patterns on their homebound journey. Physiol Entomol.

[CR4] Cheng K, Middleton EJ, Wehner R (2012). Vector-based and landmark-guided navigation in desert ants of the same species inhabiting landmark-free and landmark-rich environments. J Exp Biol.

[CR5] Cheng K, Narendra A, Wehner R (2006). Behavioral ecology of odometric memories in desert ants: acquisition, retention, and integration. Behav Ecol.

[CR6] Cheng K, Schultheiss P, Schwarz S, Wystrach A, Wehner R (2014). Beginnings of a synthetic approach to desert ant navigation. Behav Processes.

[CR7] Merkle T, Knaden M, Wehner R (2006). Uncertainty about nest position influences systematic search strategies in desert ants. J Exp Biol.

[CR8] Merkle T, Wehner R (2010). Desert ants use foraging distance to adapt the nest search to the uncertainty of the path integrator. Behav Ecol.

[CR9] Müller M, Wehner R (1994). The hidden spiral: systematic search and path integration in desert ants, *Cataglyphis fortis*. J Comp Physiol A.

[CR10] Narendra A, Cheng K, Wehner R (2007). Acquiring, retaining and integrating memories of the outbound distance in the Australian desert ant *Melophorus bagoti*. J Exp Biol.

[CR11] Pfeffer SE, Bolek S, Wolf H, Wittlinger M (2015). Nest and food search behaviour in desert ants, *Cataglyphis*: a critical comparison. Anim Cognit.

[CR12] Pfeffer SE, Wahl VL, Wittlinger M, Wolf H (2019). High-speed locomotion in the Saharan silver ant, *Cataglyphis bombycina*. J Exp Biol.

[CR13] Pfeffer SE, Wittlinger M (2016). Optic flow odometry operates independently of stride integration in carried ants. Science.

[CR14] Pfuhl G, Tjelmeland H, Biegler R (2011). Precision and reliability in animal navigation. Bull Math Biol.

[CR15] Ronacher B, Wehner R (1995). Desert ants *Cataglyphis fortis* use self-induced optic flow to measure distances travelled. J Comp Physiol A.

[CR16] Schultheiss P, Wystrach A, Legge ELG, Cheng K (2013). Information content of visual scenes influences systematic search of desert ants. J Exp Biol.

[CR17] Schultheiss P, Cheng K, Reynolds AM (2015). Searching behavior in social *Hymenoptera*. Learn Motiv.

[CR18] Sommer S, Wehner R (2012). Leg allometry in ants: extreme long-leggedness in thermophilic species. Arthropod Struct Dev.

[CR19] Steck K, Wittlinger M, Wolf H (2009). Estimation of homing distance in desert ants, *Cataglyphis fortis*, remains unaffected by disturbance of walking behaviour. J Exp Biol.

[CR20] Wehner R, Srinivasan MV (1981). Searching behaviour of desert ants, genus *Cataglyphis* (*Formicidae*, *Hymenoptera*). J Comp Physiol.

[CR21] Wehner R (1983). Taxonomie, Funktionsmorphologie und Zoogeographie der saharischen Wüstenameise *Cataglyphis fortis* (Forel 1902) Stat Nov.. Senckenbergiana Biol.

[CR22] Wehner R, Pasteels JM, Deneubourg JL (1987). Spatial organization of foraging behavior in individually searching desert ants, *Cataglyphis* (Sahara desert) and *Ocymyrmex* (Namib desert). From individual to collective behavior in social insects.

[CR23] Wehner R (2003). Desert ant navigation: how miniature brains solve complex tasks. J Comp Physiol A.

[CR24] Wehner R (2008). The desert ant's navigational toolkit: procedural rather than positional knowledge. Navigation.

[CR25] Wehner R (2009). The architecture of the desert ant’s navigational toolkit (*Hymenoptera: Formicidae*). Myrmecol News.

[CR26] Wehner R (2019). The *Cataglyphis* Mahrèsienne: 50 years of *Cataglyphis* research at Mahrès. J Comp Physiol A.

[CR27] Wittlinger M, Wehner R, Wolf H (2006). The ant odometer: stepping on stilts and stumps. Science.

[CR28] Wittlinger M, Wehner R, Wolf H (2007). The desert ant odometer: a stride integrator that accounts for stride length and walking speed. J Exp Biol.

[CR29] Wittlinger M, Wolf H (2013). Homing distance in desert ants, *Cataglyphis fortis*, remains unaffected by disturbance of walking behaviour and visual input. J Physiol Paris.

[CR30] Wolf H, Wehner R (2005). Desert ants compensate for navigation uncertainty. J Exp Biol.

[CR31] Wolf H, Wittlinger M, Bolek S (2012). Re-Visiting of plentiful food sources and food search strategies in desert ants. Front Neurosci.

[CR32] Wolf H, Wittlinger M, Pfeffer SE (2018). Two distance memories in desert ants—Modes of interaction. PLoS ONE.

[CR33] Wosnitza A, Bockemühl T, Dübbert M, Scholz H, Büschges A (2013). Inter-leg coordination in the control of walking speed in *Drosophila*. J Exp Biol.

